# Monocytes and Macrophages as Protagonists in Vascular Complications of Diabetes

**DOI:** 10.3389/fcvm.2020.00010

**Published:** 2020-02-14

**Authors:** Jenny E. Kanter, Cheng-Chieh Hsu, Karin E. Bornfeldt

**Affiliations:** ^1^Department of Medicine, University of Washington Medicine Diabetes Institute, University of Washington School of Medicine, Seattle, WA, United States; ^2^Department of Pathology, University of Washington Medicine Diabetes Institute, University of Washington School of Medicine, Seattle, WA, United States

**Keywords:** apolipoprotein C3, atherosclerosis, diabetes, cytokines, efferocytosis, monocytosis, necrotic core

## Abstract

With the increasing prevalence of diabetes worldwide, vascular complications of diabetes are also on the rise. Diabetes results in an increased risk of macrovascular complications, with atherosclerotic cardiovascular disease (CVD) being the leading cause of death in adults with diabetes. The exact mechanisms for how diabetes promotes CVD risk are still unclear, although it is evident that monocytes and macrophages are key players in all stages of atherosclerosis both in the absence and presence of diabetes, and that phenotypes of these cells are altered by the diabetic environment. Evidence suggests that at least five pro-atherogenic mechanisms involving monocytes and macrophages contribute to the accelerated atherosclerotic lesion progression and hampered lesion regression associated with diabetes. These changes include (1) increased monocyte recruitment to lesions; (2) increased inflammatory activation; (3) altered macrophage lipid accumulation and metabolism; (4) increased macrophage cell death; and (5) reduced efferocytosis. Monocyte and macrophage phenotypes and mechanisms have been revealed mostly by different animal models of diabetes. The roles of specific changes in monocytes and macrophages in humans with diabetes remain largely unknown. There is an ongoing debate on whether the changes in monocytes and macrophages are caused by altered glucose levels, insulin deficiency or insulin resistance, lipid abnormalities, or combinations of these factors. Current research in humans and mouse models suggests that reduced clearance of triglyceride-rich lipoproteins and their remnants is one important mechanism whereby diabetes adversely affects macrophages and promotes atherosclerosis and CVD risk. Although monocytes and macrophages readily respond to the diabetic environment and can be seen as protagonists in diabetes-accelerated atherosclerosis, they are likely not instigators of the increased CVD risk.

## Both Type 1 Diabetes and Type 2 Diabetes Increase the Risk of Atherosclerotic CVD

To date, almost 10% of the US population has type 1 or type 2 diabetes mellitus (T1DM or T2DM), and the prevalence of diabetes continues to rise (1–4). The prevalence of diabetes worldwide is now approaching that of the US, with over 8% of people over the age of 18 having been diagnosed with diabetes. Diabetes is classically defined as hyperglycemia, such as fasting plasma glucose ≥7 mmol/L or glycated hemoglobin (HbA1c) ≥6.5% (5). Hyperglycemia develops largely as a result of impaired insulin production and/or insulin resistance, but dyslipidemia is often also present at varying degrees in patients with poor glycemic control. This is explained in part by insulin's non-glycemic effects, including its triglyceride-lowering actions (6). Thus, relative insulin deficiency or insulin resistance results in both elevated glucose levels and lipid levels.

Both forms of diabetes significantly increase the risk of atherosclerotic cardiovascular disease (CVD). Atherosclerotic lesion morphology appears to be similar in people with T1DM and T2DM based on post-mortem studies, and both forms of diabetes increase atherosclerosis disease burden, enlarge lesion necrotic cores and increase lesional macrophage content (based on CD68 immunoreactivity), as compared with lesions from subjects without diabetes (7).

Improved glycemic control using intensive insulin therapy reduces mortality and the risk of CVD in people with T1DM (8) even after the effect of the intense insulin therapy on glucose control has waned, and over time, a significant proportion of the beneficial effects (known as the legacy effect) of intensive insulin therapy is mediated by control of lipid risk factors (9). In T2DM, improved glucose control is often not associated with protection against incident CVD, at least not in subjects with established CVD (10, 11). However, in a recent study, participants with T2DM who had been randomly assigned to intensive glucose control for 5.6 years had a lower risk of CVD events (than those who received standard therapy) during the period in which the glycated hemoglobin curves were separated. There was no evidence of a legacy effect or a mortality benefit with intensive glucose control (12). It is not known whether the effects of intense glucose control on CVD risk are due to direct effects of glucose in the artery wall.

The observed dyslipidemia associated with diabetes is characterized, not primarily by elevated low-density lipoprotein (LDL) cholesterol levels, but rather by elevated triglyceride-rich lipoproteins (TRLs)—very low-density lipoproteins (VLDL), chylomicrons, and their remnant lipoprotein particles (RLPs)—and by lower high-density lipoprotein (HDL) cholesterol and smaller denser LDL particles. This lipid profile, commonly referred to as diabetic dyslipidemia (13), is most often seen in subjects with T2DM, less well-controlled T1DM, or in subjects with T1DM with additional traits of metabolic syndrome (3).

Furthermore, recent studies suggest that apolipoprotein C3 (APOC3), an apolipoprotein that prevents clearance of TRLs and their RLPs (14–18), predicts incident CVD in subjects with T1DM even when triglyceride levels are in the normal range or close to normal range (19, 20). The effect of APOC3 as a CVD risk factor was independent of glycemic control (HbA1c levels). Therefore, changes in lipids are likely to be critical in promoting CVD risk in both T1DM and T2DM. It is, however, possible that hyperglycemia acts in concert with the lipid changes. For example, it has been suggested that hyperglycemia, due to increased formation of advanced glycation end products, leads to cross-linking of extracellular matrix molecules and arterial stiffening (21, 22), which could, in turn, exacerbate the atherogenic process driven by other factors, such as lipids. Furthermore, when present, risk factors for CVD identified in the general population, such as smoking and elevated blood pressure, exacerbate CVD risk also in people with diabetes.

The metabolic hallmarks of diabetes discussed above affect multiple cell types in the artery wall, promoting pro-atherogenic processes and progression of atherosclerosis. In particular, diabetes results in changes in the myeloid cell compartment, which includes monocytes and macrophages. In this review, we will discuss diabetes-induced alterations in monocyte and macrophage phenotypes and how these alterations might contribute to the increased CVD burden in diabetes.

## Monocytes and Macrophages Are Key Players in Diabetes-Accelerated Atherosclerosis

Monocytes and macrophages play essential roles in all stages of atherosclerosis (23), both in the presence and absence of diabetes (24). Initially, monocytes enter the subendothelial space in susceptible arteries in response to the retention of apolipoprotein B (APOB)-containing lipoproteins (25), which bind through positive charges in APOB to negatively charged extracellular matrix molecules, primarily glycosaminoglycans (26). The accumulation of monocytes in the artery wall and subsequent maturation of these cells to macrophages propagate a chronic, non-resolving low-grade arterial inflammation (27, 28). Macrophages also contribute to advanced lesions; macrophage death is largely responsible for formation and expansion of necrotic cores in advanced lesions (29). Lesions of atherosclerosis can regress in response to aggressive lipid lowering. Lesion regression is accompanied by a reduced abundance of lesional macrophages, rendering the lesion more fibrotic and likely more stable, and a pro-resolving “M2-like” macrophage phenotype (30, 31).

Early studies of the effects of diabetes on macrophages and other immune cells were pursued in part because of the interest in pancreatic immune cells contributing to diabetes development. Some of these studies suggested that diabetes suppresses macrophage functions, such as phagocytosis (32) and migration (33) in animal models of diabetes and in humans. Later studies indicated an increased activation of macrophages in diabetes (34). Different macrophage isolation methods and differences in macrophage populations might explain these discrepant findings.

Both mouse and human studies implicate macrophages as a key cell type in atherosclerosis associated with diabetes (35–37). Thus, autopsy and coronary atherectomy samples from humans have shown that lesions from people with diabetes are enriched in macrophages, as compared with specimens from patients without diabetes (38). Studies of mouse and porcine models of diabetes have revealed that arterial accumulation of glycosaminoglycans and accumulation of macrophages in early fatty streak lesions are accelerated by diabetes (39–42), consistent with an acceleration of monocyte recruitment and macrophage accumulation according to the response-to-retention hypothesis (43). Other mouse models have shown that diabetes hinders the regression of lesions through a process dependent on monocyte recruitment and skewing of the phenotype toward a more inflammatory lesional macrophage (36, 44–47). Diabetes also promotes progression of more advanced lesions in mouse and porcine models through mechanisms related to changes in macrophage abundance and phenotypes (19, 45, 46, 48–51). Thus, monocytes and macrophage are altered by the diabetic environment, and may be critical mediators of the proatherogenic effects of diabetes both in progressing and regressing lesions of atherosclerosis.

## What is the Role of Monocytosis in Diabetes-Accelerated Atherosclerosis?

Elevated levels of circulating blood monocytes (monocytosis) can result in increased monocyte recruitment to lesions, thereby accelerating lesion progression, and can also impede atherosclerosis regression. Numerous animal studies support the idea that increased monocyte levels in circulation can result in increased monocyte recruitment to the artery wall (52–54). Hypercholesterolemia is a strong driver of hematopoiesis in mice, and the early myeloid precursors in bone marrow are especially sensitive to alterations in cholesterol homeostasis (55). Recently, Nagareddy et al. in the Goldberg, Fisher and Tall laboratories suggested that these bone marrow progenitor cells also respond to signals associated with hyperglycemia (44). Previous work from this group has shown that diabetes impairs atherosclerotic lesion regression in response to dramatic lipid-lowering (36). Nagareddy demonstrated that lowering blood glucose by using a sodium-glucose cotransporter 2 (SGLT2) inhibitor in mouse models of T1DM prevented diabetes-induced monocytosis, which in turn reduced monocyte recruitment to the artery wall and improved lesion regression in diabetic mice (44). Furthermore, this interesting study suggested that rather than glucose acting directly on the bone marrow hematopoietic progenitor cell compartment, neutrophil-derived S100A8/S100A9 stimulated myelopoiesis by activating the receptor for advanced glycation end products (RAGE) on bone marrow progenitor cells (44). S100A8 and S100A9 are damage-associated molecular pattern proteins released from neutrophils and monocytes (56). S100A8 and S100A9 form dimers and exhibit both intracellular and extracellular functions. Extracellularly, S100A8/S100A9 is believed to activate RAGE or the lipopolysaccharide (LPS) receptor toll-like receptor 4 (TLR4). Subsequent studies demonstrated that the increase in neutrophil S100A8/S100A9 release in diabetic mice also causes reticulated thrombocytosis by activating RAGE on hepatic Kupffer cells, resulting in increased interleukin-6 (IL-6) production and, through increased thrombopoietin production, thrombocytosis (57). This pathway was shown to contribute to diabetes-accelerated atherosclerosis. The stimulatory effect of diabetes on thrombocytosis was also prevented by SGLT2 inhibition, suggesting that SGLT2 inhibitors prevent both diabetes-induced monocytosis and thrombocytosis in these mouse models. The same group inferred a non-glucose mediated mechanism whereby obesity/T2DM could stimulate monocytosis (58). In the obese mouse models investigated, inflamed adipose tissue rather than neutrophils appears to be the primary source of S100A8/S100A9. S100A8/S100A9 prompts adipose tissue macrophages to release interleukin-1β (IL-1β) through a TLR4-dependent mechanism, which in turn stimulates myelopoiesis by acting on bone marrow progenitors (58).

Together, these studies demonstrate that monocytosis is associated with exacerbated atherosclerosis in diabetic mouse models and that the damage-associated molecular pattern molecules S100A8 and S100A9 play a critical role in this process. Similar mechanisms might be relevant in humans because plasma S100A8/S100A9 levels were shown to correlate with leukocyte counts and coronary artery disease in patients with T1DM (44) and to be increased in patients with T2DM (57). Furthermore, in a mouse model of T1DM, increased intraplaque hemorrhage in advanced lesions was shown to correlate with increased lesional S100A9 immunoreactivity (48). Very recently, diabetes-induced myelopoiesis, monocytosis, neutrophil production of S100A8/S100A9 and impaired lesion regression were found to be prevented by the increased levels of HDL in a human APOA1 transgenic mouse model (47), suggesting that diabetes-induced monocytosis and hampered lesion regression can be prevented by increasing cholesterol efflux from myeloid cells and their bone marrow progenitors.

However, diabetes is not consistently associated with monocytosis in mouse models, and monocytosis is not routinely shown to be present in people with diabetes. Furthermore, in a recent study, SGLT2 inhibition accelerated atherosclerosis regression in diabetic mice without altering circulating monocyte levels (59), demonstrating that SGLT2 inhibition does not act solely by reducing monocytosis in mouse models. In this study, SGLT2 inhibition prevented leukocyte adhesion to the artery wall as well as lesional macrophage proliferation, rather than affecting monocytosis. In mouse models, the ability of diabetes to induce monocytosis appears to be dependent on the diet. Thus, our studies have shown that hyperglycemic mouse models of T1DM fed a low-fat semi-purified diet do not exhibit monocytosis, yet show accelerated atherosclerosis (19, 60). In obese hyperlipidemic mouse models of T2DM or metabolic syndrome (pre-diabetes), on the other hand, monocytosis is consistently present and is likely to contribute to the accelerated atherosclerosis in these mice (61, 62). These findings are consistent with the observations that cholesterol promotes monocytosis by acting on bone marrow myeloid cell precursors (55) and that diabetes-induced myelopoiesis can be prevented by increasing cholesterol efflux through elevated HDL levels (47).

Together, these studies suggest that diabetes-induced monocytosis can contribute to, but is not necessary for, diabetes-accelerated atherosclerosis in mouse models. Thus, additional mechanisms are important in driving diabetes-accelerated atherosclerosis both in mouse models and in humans.

## Does Increased Inflammatory Activation of Monocytes and Macrophages Contribute to Diabetes-Accelerated Atherosclerosis?

Monocytes enter peripheral tissues in response to tissue changes the body perceives as an injury, such as atherosclerosis. Once in tissues, these cells differentiate into macrophages. Macrophages are highly plastic cells that take on different phenotypes depending on their surrounding micro-environment. Their primary roles are to phagocytose pathogens and dead and dying cells, which contributes to healing of the perceived injury, and to produce chemokines and cytokines to call in additional leukocytes to aid in the protection of the organism when needed. Macrophages thereby contribute to both the pro-inflammatory and the pro-resolution phases of inflammation.

**Figure 1:**
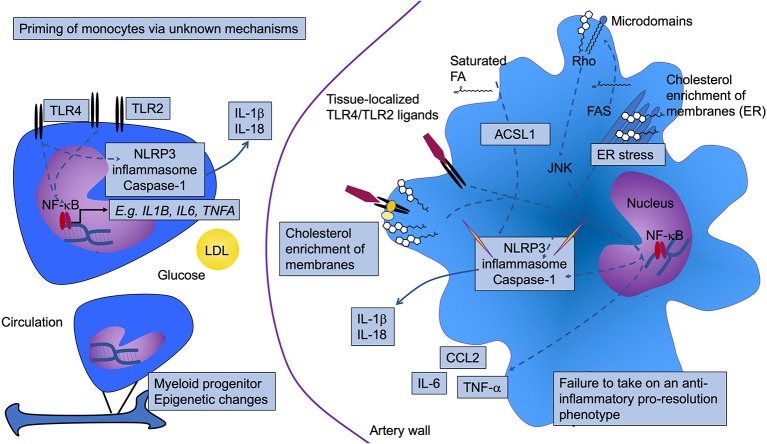
Diabetes is associated with increased monocyte and macrophage inflammatory status. Diabetes results in activation and priming of monocytes to take on a more inflammatory phenotype. Although the mechanism whereby the priming occurs is incompletely understood, glucose, low-density lipoprotein (LDL), and epigenetic modifications have been implicated. This phenotype includes increased gene expression of prototypical pro-inflammatory cytokines, such as IL-1β, TNF-α, and IL-6 (likely through activation of NF-κB), as well as increased expression of toll-like receptors (TLR) 2 and TLR4. Once the monocytes enter the artery wall and differentiate into macrophages, they also display a heightened inflammatory status. The elevated TLR2/4 levels may interact with tissue-localized TLR ligands. Alternatively, or in addition, the aberrant lipid loading observed in diabetes might result in changes in membrane cholesterol accumulation, resulting in increased proximity of TLRs with other proteins that enhance TLR signaling. Increased cholesterol and saturated fatty acids (FA), either through altered cellular membrane properties, as crystals, and/or as inducers of ER stress, can activate the NLRP3 inflammasome and caspase 1, thereby contributing to increased inflammation through release of IL-1β and IL-18. In human monocytes, TLR4 activation is sufficient for inflammasome and caspase 1 activation, as indicated by a direct arrow from TLR4 to the NLRP3 inflammasome and caspase 1. ACSL1 (long-chain acyl-CoA synthetase 1) is induced in inflammatory macrophages. This enzyme acts on free fatty acids to produce acyl-CoA species, which in the case of saturated acyl-CoAs have been shown to contribute to lipotoxicity and inflammasome activation. Fatty acid synthase (FAS) might also contribute by generating fatty acid substrates that are then incorporated into glycerolipids in the membrane where membrane microdomains regulate signaling via Rho activation of JNK. Pathways that have been shown to be altered by diabetes are highlighted by boxes. NLRP3, NOD-like receptor protein 3.

Many studies have demonstrated that diabetes causes monocytes to take on a more inflammatory phenotype, both in mouse models and in humans. For example, isolated CD14-positive human peripheral blood monocytes from subjects with T1DM or T2DM express and produce elevated levels of the pro-inflammatory cytokines IL-1β, tumor necrosis factor-α (TNF-α), and IL-6, as compared to monocytes isolated from control subjects (63–66). Likewise, blood monocytes from a mouse model of T1DM exhibit elevated levels of *Tnfa* mRNA (37). In addition to increased cytokine production under basal conditions, several groups have suggested that monocytes isolated from subjects with diabetes are “primed” to respond more robustly, as compared with monocytes isolated from controls, to inflammatory stimuli, such as LPS or interferon-γ. The increased expression of TLRs, such as TLR2 and TLR4, on the cell surface of monocytes from people with diabetes supports this hypothesis (63–65, 67). It is possible that the primed state of monocytes results in a heightened inflammatory activation once the monocytes encounter TLR ligands in the lesion (Figure 1).

Post-mortem studies of atherosclerotic lesions from both subjects with T1DM and T2DM suggest that diabetes increases the abundance of CD68-positive lesion cells likely to be macrophages (7, 38). To date, there are no studies on macrophage function or phenotype in response to diabetes in human atherosclerotic lesions. However, isolated CD68-positive macrophages from chronic wounds suggest that diabetes results in a less anti-inflammatory and a more pro-inflammatory macrophage phenotype, characterized by reduced expression of CD206 (a pattern recognition receptor that can recognize microbial carbohydrates) and CD163 (a scavenger receptor for the hemoglobin-haptoglobin complex), which are often used as markers of the alternatively activated “M2” macrophage phenotype, and increased expression of the pro-inflammatory cytokine IL-12 upon *ex vivo* stimulation (68). Whether similar changes also occur in human lesional macrophages in response to diabetes is unknown.

Studies in obese hyperglycemic leptin receptor-deficient mice (a model of T2DM) imply that diabetes impairs the resolution of the inflammatory response during wound healing, with macrophages from diabetic mice continuing to produce pro-inflammatory markers even during the resolution phase (68, 69). At least in mice, similar processes transpire in atherosclerotic lesions in response to aggressive lipid-lowering, in which CD68-positive cells in diabetic mice fail to undergo a switch to a less inflammatory and more “M2-like” pro-resolution macrophage (36, 44). Under conditions of atherosclerotic lesion regression in response to aggressive lipid-lowering, a large proportion of the reduction in lesional macrophage accumulation could be attributed to reduced monocyte recruitment together with a stable rate of apoptosis of plaque macrophages [(70); Figure 1]. Interestingly, work from the Fisher laboratory demonstrated that lesion regression appears to require recruitment of the Ly6C^hi^ monocyte population, and the conversion of these monocytes to “M2-like” alternatively activated macrophages within the lesion (30). This pathway appears to be impaired by diabetes. The mechanism whereby alternatively activated or pro-resolving macrophages contribute to lesion regression is still unclear. One possibility is that these macrophages are more effective in clearing dying or dead cells through efferocytosis than are inflammatory macrophages (71).

Interestingly, at least in mice, the changes observed in macrophages during wound healing are driven by epigenetic modifications that occur in the hematopoietic compartment (68, 69). The notion that diabetes results in lasting effects on the myeloid cell compartment are further highlighted by the fact that if blood monocytes are isolated from T2DM subjects or matched controls, and then differentiated into macrophages for 5 days *in vitro*, monocyte-derived macrophages from subjects with diabetes express elevated levels of numerous pro-inflammatory cytokines, including *IL1B, CCL2*, and *IL6*, as compared with monocyte-derived macrophages from controls (72). Using a similar model, it was shown that monocyte-derived macrophages from patients with T2DM exhibit increased expression of NLRP3 and inflammasome activation [(72); Figure 1].

The factors responsible for monocyte priming in diabetes are largely unknown. One study showed that the priming can be mimicked *in vitro* by a combination of LDL and high glucose and appears to be mediated by the redox-sensitive MAPK phosphatase MKP-1 (73). Furthermore, epigenetic changes have recently been shown to be responsible for a phenomenon called “trained immunity,” which allows innate immune cells to mount a response to reinfection (74). It has been suggested that diabetes might induce epigenetic changes in monocytes through trained immunity [(75); Figure 1], although research is needed to provide evidence for this hypothesis.

The recent Canakinumab Antiinflammatory Thrombosis Outcome Study (CANTOS) indicates a causative role for IL-1β in atherosclerotic cardiovascular disease in humans (76). Subjects who had already had a myocardial infarction and had elevated levels of high-sensitivity C-reactive protein were treated with a blocking antibody to IL-1β, and cardiovascular death, myocardial infarction, or stroke were evaluated. There was a significant reduction in the rate of CVD in those treated with the IL-1β blocking antibody, demonstrating for the first time a role for inflammation in CVD in humans in these high-risk subjects (76). Interestingly, subjects with and without diabetes within the trial had a similar reduction in major cardiovascular events (77). This might suggest that inflammation is not the culprit in the diabetes-acceleration of atherosclerosis. Alternatively, it might merely indicate that when comparing diabetes to other states of heightened inflammation (per inclusion criteria for this trial), there is no further role for inflammation associated with diabetes.

A very recent single-cell RNA-sequencing study describes the transcriptome of leukocytes from asymptomatic and symptomatic human carotid atherosclerotic lesions. Strikingly, the authors found that a TLR4 and IL-1β signaling signature was associated with macrophages from asymptomatic lesions, rather than symptomatic lesions (78). Instead, macrophages in symptomatic lesions were characterized by an alternatively activated signature, including IL-4-mediated signaling, suggesting a healing function. This finding indicates that macrophage phenotypes in symptomatic lesions are not geared to inflammation but rather to healing, whereas macrophages in lesions that have not yet caused symptoms harbor more active inflammation. These types of studies have not yet been done in people with diabetes.

In summary, there is evidence that diabetes promotes a more inflammatory monocyte and macrophage phenotype, and that the heightened inflammatory capacity of macrophages might be facilitated by priming of blood monocytes or potentially of myeloid progenitor cells by the diabetic environment (Figure 1). It is not known to what extent the increased inflammatory capacity of monocytes and macrophages directly contributes to atherosclerosis progression in diabetes.

## Aberrant Lipid Metabolism in Monocytes and Macrophages is a Critical Component of Diabetes-Accelerated Atherosclerosis

**Figure 2:**
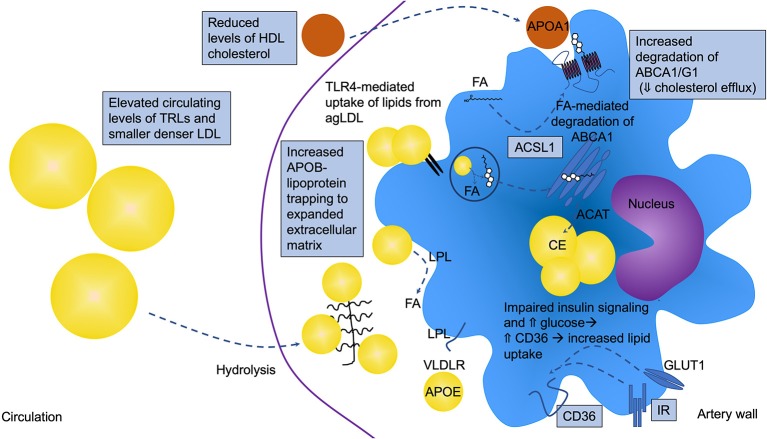
Altered macrophage lipid handling is critically involved in diabetes-accelerated atherosclerosis. Under diabetic conditions, macrophages are often lipid loaded and contain increased levels of cholesteryl ester (CE) in lipid droplets. T2DM and poorly controlled T1DM are associated with elevated circulating levels of triglyceride-rich lipoproteins (TRLs), which contribute to macrophage lipid loading, but the increased lipid loading can also be in part due to increased retention of atherogenic lipoproteins in the artery wall, such as LDL, VLDL, and remnant lipoprotein particles (RLPs). These APOB-containing lipoproteins can bind the expanded/altered extracellular matrix present in lesions in diabetes. Diabetes also alters the balance between uptake and efflux of lipids by modulating the levels of receptors and transporters involved in cholesterol and fatty acid uptake and efflux. CD36 and very low-density lipoprotein receptor (VLDLR), in addition to other receptors and mechanisms, mediate lipid uptake while ATP-binding cassette transporter A1 and G1 (ABCA1 and ABCG1) are critical mediators of cholesterol efflux to HDL and APOA1. ABCA1 and CD36 have been shown to be reduced and elevated, respectively, under diabetic conditions. Fatty acids have been demonstrated to mediate increased degradation of ABCA1 through a mechanism dependent on ACSL1. Impaired insulin signaling via the insulin receptor (IR) and elevated glucose can lead to increased CD36 levels. Furthermore, the elevated levels of TLR4 might result in increased uptake of lipids from aggregated LDL (agLDL), further contributing to the increased lipid loading. Lipoprotein lipase (LPL) aids in the hydrolysis of TRLs, generating free fatty acids (FA) that are easily taken up by cells. All of these pathways can be altered by the diabetic environment as indicated in the schematic by boxes, resulting in lipid accumulation and a pro-atherogenic macrophage phenotype.

Macrophages in lesions of atherosclerosis will engulf APOB-containing lipoprotein particles (LDL, VLDL, and RLPs), resulting in the formation of the hallmark cell type of atherosclerosis—foam cells. Large TRLs, such as chylomicrons derived from the gut after a meal, are believed to be too large to enter the artery wall without first being partly hydrolyzed to RLPs by lipoprotein lipase. While LDL needs to be modified, e.g., by oxidation, to be effectively ingested by macrophages, VLDLs and RLPs are readily taken up by macrophages without modification (79, 80). Early studies showed that VLDL isolated from patients with T2DM is taken up more effectively by mouse peritoneal macrophages, and results in increased lipid accumulation, as compared with VLDL from subjects without diabetes (81). In addition to macrophages, other cell types significantly contribute to foam cell formation in lesions, such as smooth muscle cells, which are known to transdifferentiate into macrophage-like cells after they become lipid-laden (82). Although the effect of diabetes on smooth muscle cell lipid loading and subsequent transdifferentiation to macrophage-like cells is unknown, it has been shown that macrophages become more loaded with cholesteryl esters in the presence of diabetes (19). This might be due in part to increased levels of RLPs in the interstitial fluid surrounding macrophages in the setting of diabetes [(19); Figure 2]. Diabetes has also been shown to lead to reduced expression of the cholesterol transport proteins ABCA1 and ABCG1 in macrophages (83–85), which would reduce cholesterol efflux and further increase their cellular cholesterol content. Furthermore, high glucose and reduced insulin signaling have been suggested to increase the expression of CD36, a lipid scavenger receptor, in macrophages and monocytes through post-transcriptional mechanisms (86, 87). Together, these observations demonstrate that macrophages carry a larger load of lipid in the presence of diabetes (Figure 2).

It was long believed that the uptake of lipoproteins invariably results in an increased pro-inflammatory state of macrophages (88–90). However, recent studies indicate the opposite is true, at least under some circumstances, perhaps when the macrophage's ability to handle the increased lipid load is unharmed (91, 92). Accordingly, Kim et al. recently demonstrated that foamy macrophages isolated from atherosclerotic lesions had an attenuated inflammatory repertoire. In contrast, the non-foamy macrophages expressed elevated levels of pro-inflammatory markers and overlapped with the inflammatory macrophage cluster identified by single-cell RNA sequencing (92). Along the same lines, we recently demonstrated that macrophage lipid loading and their inflammatory capacity do not go hand-in-hand in the setting of diabetes (19). We observed, by using a mouse model of T1DM, that diabetes results in both heightened inflammation and increased cholesteryl ester loading in cells isolated from the peritoneal cavity. However, when the diabetic mice were treated with an antisense oligonucleotide (ASO) to APOC3 *in vivo*, the increased macrophage cholesteryl ester loading associated with diabetes was prevented while the heightened *Il1b* and *Tnfa* gene expression was unaffected (19). Moreover, diabetes-accelerated atherosclerosis closely associated with the prevention of macrophage cholesteryl ester accumulation and was dramatically reduced by the APOC3 ASO, suggesting that reduced lipid loading of macrophages might be more important for atherosclerosis than inflammatory changes in these cells in diabetes.

**Figure 3:**
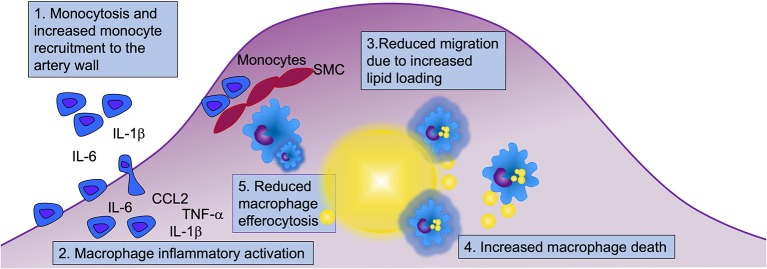
Myeloid cells contribute to diabetes-accelerated atherosclerosis and hindered lesion regression through at least five mechanisms. Diabetes alters many aspects of myeloid cell biology that can contribute to accelerated atherosclerosis and hampered lesion regression. Both monocytes and macrophages display an augmented inflammatory potential, and under certain conditions, diabetes results in elevated levels of monocytes in circulation, which when present contribute to increased recruitment of monocytes to the artery wall. Diabetes increases macrophage lipid loading (indicated by yellow lipid droplets in blue macrophages), which in turn might reduce their ability to migrate. Lipid overload may drive macrophage cell death, and together with reduced efferocytosis, this is likely to contribute to the acceleration of necrotic core formation and progression of atherosclerosis in diabetes. Five mechanisms involving monocytes and macrophages that are likely to contribute to diabetes-accelerated atherosclerosis are highlighted (numbered in boxes). SMC, smooth muscle cells.

Macrophage lipid loading results in an impaired migratory capacity, which could contribute to trapping of the macrophage within the lesion [(93, 94); Figure 3]. Work from the Randolph laboratory suggests that macrophages within the atherosclerotic lesion have a limited migratory capacity (95) and that macrophages are stagnant cells with limited mobility once recruited to lesions, especially if the cells display a morphology consistent with lipid-laden cells. Smaller cells nearer the shoulder region and surface of the plaque exhibit increased movement; however, when monocytes were labeled at different time-points, there was no overlap in the macrophages being labeled, suggesting little movement of the macrophages once they were located deeper in the lesion. This important experiment also showed that monocytes accumulate in waves and implied that there is little phagocytosis of older macrophages by newly infiltrating cells (95). Lipid loading extends beyond macrophages; monocytes have been shown to accumulate lipid droplets, especially if they encounter TRLs, which in turn impair their migration (96). Our recent findings suggest that diabetes increases the ensnaring of pro-atherogenic particles containing APOB, APOC3, and APOE (indicating that they might be TRLs or RLPs derived from TRLs) in the artery wall and that this contributes to accelerated atherosclerosis (19). The extracellular matrix, which is responsible for retention of these lipoprotein particles in the artery wall, is expanded and altered under diabetic conditions (39, 42), and VLDL and LDL particles enriched in APOC3 bind better to matrix proteins (97), potentially indicating that under diabetic conditions, macrophages encounter more lipids within the artery wall (Figure 2).

There are, however, several circumstances in which lipid handling goes awry and lipids promote inflammatory activation of macrophages (Figure 1). One such mechanism is NLRP3 inflammasome activation by cholesterol crystals, which are present in lesions of atherosclerosis (98). A similar mechanism has been proposed for crystals generated by saturated fatty acids (99). The extent to which cholesterol- or saturated fatty acid crystals activate macrophages *in vivo* still needs to be investigated, as some have cautioned that the crystalline material may have been formed during sample processing due to the crystallization of lipids in refrigerated samples (100). The NLRP3 inflammasome is a multimeric complex that processes pro-IL-1β and pro-IL-18 into their mature forms. The NLRP3 inflammasome typically requires two signals—a priming step depending on TLR activation that induces the transcription of *Nlrp3* and *Casp1* (caspase 1)—and an activating step resulting in the assembly and activation of caspase 1 and subsequent processing of pro-IL-1β and pro-IL-18 to mature IL-1β and IL-18 (101). The activation step can be achieved by crystalline materials, ion flux induced e.g., by ATP, and ER stressors. Interestingly, human monocytes, as opposed to human macrophages, release processed IL-1β after a one-time stimulation of TLRs (102). This was explained by the finding that these cells also release endogenous ATP (102), or that an alternative inflammasome pathway exists in these cells (103). The ATP released from newly recruited monocytes might therefore contribute to inflammasome activation in human lesional macrophages by providing ATP as an activating signal.

Many groups have reported that diabetes results in increased expression of genes that would be consistent with increased inflammation and with priming/and or activation of the NLRP3 inflammasome in both T1DM and T2DM (36, 37, 44, 58, 66, 104–109) (see the above section), but it is not yet clear what the diabetes signal that drives augmented NLRP3 activation might be. It is possible that increased intracellular cholesterol crystals, crystals formed by saturated fatty acids, or altered membrane levels of cholesterol or saturated fatty acids might explain this phenomenon. Aberrant intracellular cholesterol handling can also trigger the inflammasome via increased ER cholesterol accumulation [(100, 110, 111); Figure 1]. In diabetes, the aberrant cholesterol accumulation in membranes might be due to reduced expression of the cholesterol transport proteins ABCA1 and ABCG1 in macrophages (83–85). These transporters efflux cholesterol to APOA1 and HDL, thereby reducing cellular and membrane cholesterol levels (Figure 2). The ER membrane appears to serve as an important sensor of intracellular cholesterol homeostasis (112). Some, but not all studies, have found that deletion of the inflammasome reduces atherosclerosis under non-diabetic conditions (63, 98, 100, 113, 114). Further studies are needed to investigate the role of the NLRP3 inflammasome in macrophages in the setting of diabetes.

Based primarily on studies of macrophages deficient in ABCA1 and ABCG1, in which cholesterol loading is particularly high due to the defective cholesterol efflux, it has been shown that TLR4 signaling is enhanced by cellular cholesterol accumulation. The mechanism is believed to be due to increased activation of TLR4 due to increased proximity of TLR4 with its binding proteins MD2 and CD14 in lipid rafts [(115); Figure 1]. Furthermore, a recent study suggested a different role of TLR4 in relation to macrophage lipid loading. This study demonstrated that TLR4 is required for macrophage lipid uptake from aggregated LDL, by promoting the release of exosomal enzymes for degradation of lipids in these aggregates (116). Accordingly, TLR4-deficiency reduced lipid loading of macrophages through a pathway involving PI3K and Akt (Figure 2). Direct evidence for these pathways in macrophages in diabetes is missing, but the models would fit with the increased inflammatory activation and lipid loading of these cells in diabetes.

In addition, increased fatty acid load is likely to contribute to macrophage activation through several mechanisms. Recent studies have revealed that fatty acid synthesis through fatty acid synthase (FAS) is required for macrophage inflammatory activation (117). Loss of FAS altered membrane order and composition, impairing the retention of plasma membrane cholesterol and disrupting Rho GTPase trafficking, resulting in impaired cell adhesion, migration, and activation (117). Interestingly, the effect of FAS-deficiency could be reversed by the addition of cholesterol, suggesting that endogenous fatty acids are critical for correct membrane cholesterol localization (Figure 1).

Disruption of fatty acid storage in inert triacylglycerol is associated with saturated fatty acid-induced inflammatory changes in macrophages (118). However, lipid uptake via the VLDL receptor (VLDLR) results in pro-inflammatory effects in macrophages concomitant with both increased triacylglycerol and C16:0 ceramide levels (119). VLDLR binds APOE, rather than APOB, and acts in concert with LPL to promote uptake of lipids from APOE-containing VLDL, RLPs and chylomicrons (120). Furthermore, we and others have demonstrated that deletion of acyl-CoA synthetase 1 (ACSL1), an enzyme that esterifies fatty acids into their acyl-CoA derivatives, a step necessary for most downstream fatty acid metabolism, reduces inflammation and lipotoxicity in response to saturated fatty acids and diabetes (37, 121, 122). Moreover, high levels of unsaturated fatty acids (C18:1 and C18:2) induce degradation of ABCA1, reducing cholesterol efflux capacity through a mechanism mediated by ACSL1 in macrophages (123). Myeloid cell-targeted deletion of ACSL1 also prevented diabetes-accelerated atherosclerosis (37), demonstrating the important role of fatty acid metabolism in atherosclerosis in the setting of diabetes (Figures 1, 2).

Together, these studies indicate that aberrant lipid accumulation and handling in macrophages can cause, or is associated with, increased inflammation. Prevention of macrophage lipid loading in diabetic mice associates with prevention of accelerated atherosclerosis (19). Further studies are needed in order to clarify the effects of diabetes on macrophage cholesterol and fatty acid handling, the relationships among lipids and inflammation, and the relative impacts of these pathways on atherosclerosis.

## Diabetes Exacerbates Lesion Progression and Necrotic Core Expansion

Human imaging studies suggest that diabetes accelerates lesion progression. Thus, Won et al. used coronary computed tomography angiography to demonstrate that atherosclerotic lesion progression is faster in people with diabetes than in those without, despite having lower LDL cholesterol at baseline (124). In a similar study, Kim et al. demonstrated that plaque progression (necrotic core expansion), was accelerated in diabetes—again in the absence of elevated LDL cholesterol (125). These studies highlight the concept that diabetes accelerates atherosclerotic lesion progression even in settings in which LDL cholesterol levels are lower or similar as compared with the non-diabetic control cohort. In both the aforementioned studies, mean LDL cholesterol levels were ~100 mg/dL, which is in the normal range. Mouse studies of diabetes-accelerated atherosclerosis also support this concept (39).

As the atherosclerotic lesion progresses, a necrotic core is formed. Necrotic core expansion is thought to be due, at least in part, to the death and reduced clearance of dead and dying macrophages (Figure 3). Both human pathology studies and imaging studies have demonstrated that diabetes results in an expansion of lesional necrotic cores associated with lesion progression (126–129). Necrotic core expansion can also be observed in mouse models of T1DM (19) and of T2DM/metabolic syndrome (61, 130). It is unclear to what extent the expanded necrotic cores observed in diabetes are due to a selective modulation of the myeloid cell compartment, resulting in increased macrophage death or reduced clearance of dead and dying macrophages, or if the expanded necrotic core is a marker of a faster-progressing lesion, which might be due to increased recruitment and turnover of macrophages (Figure 3). Alternatively, all three scenarios could be occurring. For example, deletion of insulin receptors in myeloid cells results in increased susceptibility of macrophages to apoptosis in response to altered cholesterol metabolism, causing larger necrotic cores in mouse models [(131); Figure 3]. Thus, insufficient insulin signaling might contribute directly to increased macrophage death and necrotic core expansion. Furthermore, if there indeed is increased inflammasome activation in diabetes, this can result in pyroptosis, an inflammatory type of cell death linked to the NLRP3 inflammasome that has been proposed to play a role in atherosclerosis (132). Future research will reveal whether pyroptosis or other cell death pathways are increased in macrophages in diabetes and if such mechanisms contribute to diabetes-accelerated lesion progression.

The clearance of dying or dead cells by phagocytes is termed efferocytosis. Efferocytosis has been shown to be impaired in mouse models of T2DM, an effect ascribed to the increased membrane content of saturated fatty acids (130). Reduced efferocytosis can contribute to the expansion of necrotic cores and the progression of the atherosclerotic lesion [(133); Figure 3].

Efferocytosis requires metabolic reprogramming to increase glucose uptake and aerobic glycolysis (134). A prevailing hypothesis in the field of diabetes is that if a cell is bathed in excessive glucose, it ought to take up more glucose, especially if the cell expresses non-insulin dependent glucose transporters, such GLUT1. Macrophages express relatively high levels of GLUT1 and lower levels of the insulin-sensitive GLUT4. However, because GLUT1 exhibits a K_m_ for glucose of 1–7 mM, it is generally believed to be nearly saturated at physiological ranges of glucose (135). Instead, increased glucose influx through GLUT1 might be a result of inflammatory activation of macrophages (136). To investigate if increased glucose flux into myeloid cells *per se* could mimic the effects of diabetes in non-diabetic mice, we overexpressed GLUT1 under control of the CD68 promoter in hematopoietic cells, which results in expression primarily in myeloid cells (136). Forcing macrophage to take up more glucose through GLUT1 overexpression did not induce inflammatory changes in primary macrophages *ex vivo* or accelerate atherosclerosis or alter necrotic core size (136). However, deletion of GLUT1 prevented normal inflammatory activation of these cells by LPS or LPS and interferon-γ (136, 137). Thus, GLUT1 is required for normal inflammatory activation of macrophages, consistent with previous studies (138), but increased glucose flux through GLUT1 is not sufficient to promote lesion progression in non-diabetic mice. However, if GLUT1 is deleted selectively in myeloid cells in non-diabetic mice, efferocytosis is reduced, and the necrotic core area is expanded, because of the reliance on glucose metabolism to carry out phagocytosis and efferocytosis (134, 137). Conversely, GLUT1 deletion from the entire hematopoietic compartment results in reduced myelopoiesis in response to dyslipidemia, reduced monocyte recruitment to the artery wall, and reduced atherosclerosis (139). Together, these three studies highlight the complexity of glucose metabolism within different closely related cell types. Increased glucose uptake through GLUT1 in myeloid cells does not appear to be sufficient for inflammatory activation or lesion progression, but GLUT1 is required for several functions of myeloid cells and their precursors.

The findings to date suggest that diabetes enhances necrotic core formation in lesions, perhaps because it promotes lesion progression to more advanced lesions, and possibly in part because it has direct effects on macrophage death and adverse effects on efferocytosis. The effects of diabetes on necrotic core expansion do not appear to be due to increased glucose uptake in macrophages, but rather to be dependent on a reduction in efferocytosis due to increased macrophage membrane saturation (130), and perhaps to increased macrophage death due to insufficient insulin signaling or other mechanisms. Lesions with large necrotic cores are believed to be responsible for a significant proportion of the clinical events of CVD, being less stable and more susceptible to fissuring or rupturing.

## Are Kidney Macrophages in Diabetic Kidney Disease Altered in Ways Similar to Lesional Macrophages in Atherosclerosis?

Since monocytes are circulating cells that enter all injured tissues, do some of the changes observed in lesional macrophages also occur in other tissues affected by diabetes complications? In addition to macrovascular disease, diabetes increases the risk of microvascular complications, such as diabetic retinopathy and diabetic kidney disease (DKD). Several lines of evidence suggest that the presence of microvascular disease dramatically increases the risk of macrovascular complications (140–142). Whether this is due to a shared underlying mechanism or if one complication accelerates the other is unknown. If diabetes influences the myeloid cell compartment and alters monocyte and macrophage function as it relates to atherogenesis, perhaps changes in monocytes and macrophages also contribute to other complications of diabetes, such as DKD. Such changes in monocytes and macrophages might, in part, explain why DKD and atherogenesis are tightly linked.

Do monocytes and macrophages play a causative role in DKD? Macrophages are known to accumulate in the glomerulus and renal interstitium in kidney disease (143–145). Monocyte depletion studies in preclinical models suggest that monocytes and macrophages play a direct pathological role in DKD, perhaps by affecting podocyte barrier function (146). Similarly, deletion or reduction of CCL2, a key chemokine involved in monocyte recruitment, reduces diabetic kidney disease in mice (147–149). More recently, two clinical trials targeting CCL2 have been completed with promising improvements of albuminuria in humans with T2DM and kidney disease (150, 151). Very recently, Niewczas et al. highlighted the importance of chronic inflammation in the progression of kidney disease in diabetes (152). A cluster of 17 kidney disease risk signature proteins that include TNF family proteins and several cytokines predicted the risk of end-stage renal disease, with a least some of these proteins being produced in the glomerulus, and some of them correlating with histopathological changes associated with diabetes, such as glomerulosclerosis (152). This network included the IL-1 receptor, which might suggest activation of the inflammasome pathway.

Lipids accumulate in glomeruli in DKD (153), consistent with the reduced ABCA1 expression in kidneys from diabetic mice (83). TNF-α induces cholesterol accumulation in cultured podocytes, which in turn exacerbates apoptosis in these cells (154), suggesting links among lipid accumulation, lipotoxicity, and inflammatory stimuli. Consistent with this notion, dyslipidemia associated with atherosclerosis augmented albuminuria in a mouse model of DKD (155). Interestingly, the pro-atherogenic dyslipidemia was associated with increased glomerular macrophage accumulation and a dramatic increase in renal cortex inflammation, suggesting that dyslipidemia contributes to DKD, perhaps via augmented macrophage activation analogous to that in the artery wall. Further research is needed to address the links among diabetes-induced macrophage phenotypic changes and DKD and other diabetes complications.

## Do Anti-Diabetic Medications Shown to be Effective in Preventing CVD Risk Alter Monocyte and Macrophage Phenotypes?

In 2008, the Food and Drug Administration recommended to include cardiovascular endpoints in all phase 2 and phase 3 clinical trials of new anti-diabetic therapies. As a result of this recommendation, new anti-diabetic therapeutics were revealed to have unexpected protective effects on cardiovascular outcomes. Glucagon-like peptide 1 (GLP-1) receptor agonists, which increase insulin secretion, and SGLT2 inhibitors, which lower blood glucose levels by preventing glucose reabsorption in the kidney, both were shown to reduce cardiovascular events in patients with T2DM (156–160). GLP-1 receptor agonists have multiple beneficial effects in several organs in humans, resulting in reduced risk of atherosclerotic CVD events and CVD death. Among the many beneficial effects of GLP-1 receptor activation are anti-inflammatory effects, direct effects in the heart, increased natriuresis and diuresis, reduced body weight, reduced coagulation, and a reduction in post-prandial lipids, as reviewed extensively elsewhere (161). SGLT2 inhibitors display the greatest beneficial effect on heart failure and progression of renal disease, and have only modest beneficial effects on reducing atherosclerotic CVD, and primarily are effective in patients with established atherosclerotic disease (162). Furthermore, SGLT2 inhibitors exert beneficial actions independent of glucose control, consistent with results from the recent DAPA-HF (Dapagliflozin and Prevention of Adverse Outcomes in Heart Failure) trial, which indicated beneficial effects of this SGLT2 inhibitor (dapagliflozin) in patients with heart failure and a reduced ejection fraction even when diabetes was not present (163). Suggested beneficial mechanisms not directly related to blood glucose-lowering effects include natriuresis and osmotic diuresis, reduced inflammation, reduced oxidative stress, reduced arterial stiffness, reductions in blood pressure and body weight, and renoprotective effects (164). According to the safety profiles, SGLT2 inhibitors do not appear to reduce monocyte counts in humans with T2DM, suggesting that the effects of SGLT2 inhibitors on monocytosis in diabetic mice might not be translated to humans. Other clinical trials have demonstrated beneficial effects on CVD outcomes of icosapent ethyl ester, a highly pure fish oil ester, in cohorts with and without T2DM (165, 166).

Do these drugs have direct effects on monocytes and macrophages and is it possible that some of the beneficial effects are due to alterations of lesion macrophages? SGLT2 inhibitors have been shown to prevent macrophage foam cell formation in diabetic mice concomitant with reduced atherosclerosis (46). The mechanism was suggested to be due to downregulation of the increased LOX1 and ACAT and upregulation of the decreased ABCA1 in macrophages from diabetic mice treated with the SGLT2 inhibitors. Overall however, current research suggests that the main therapeutic effects of SGLT2 inhibitors are unlikely to be mediated by effects in myeloid cells, at least in humans.

GLP-1 receptor agonists prevent incident atherosclerotic CVD in people with diabetes. Some of the effects might be mediated by direct effects on macrophages, although GLP-1 receptor agonists exert effects in a plethora of cell types, and macrophages are unlikely to be a major point of action. Nevertheless, exendin-4 (a GLP-1 receptor agonist) has been shown to reduce atherosclerosis and monocyte adhesion to aortas as well as inflammatory gene expression in macrophages in mice, likely by increasing cAMP levels in these cells (167).

In relation to the beneficial effects of icosapent ethyl ester on incident CVD, fish oils have been shown to have beneficial effects in macrophages, including reversal of the defective efferocytosis associated with diabetes (130). Although the mechanism of icosapent ethyl ester might be distinct from that of natural mixed fish oils, it is relevant that fish oils can be converted to bioactive lipid mediators involved in the resolution of inflammation (168).

More research is needed to understand the beneficial effects of these therapies in patients with diabetes. Such studies could also shed additional light on the mechanisms whereby diabetes increases CVD risk.

## Summary and Future Questions

Diabetes creates an environment that fosters the initiation and progression of the atherosclerotic lesion as well as a hampered lesion regression through at least five likely mechanisms in monocytes and macrophages (Figure 3). First, diabetes increases monocyte recruitment to the lesion, especially in the setting of monocytosis. However, while monocytosis can exacerbate monocyte recruitment to lesions, it is not required for diabetes-accelerated atherosclerosis. Second, monocytes appear to be primed in circulation to have an increased inflammatory potential, and once in the artery wall, their boosted cytokine/chemokine production can contribute to increased recruitment of new monocytes and activation of macrophages and other lesional cells. Third, diabetes increases macrophage lipid loading, perhaps mainly due to trapping of TRLs and RLPs or modified LDL within the artery wall via increased retention of these lipoprotein particles through binding to glycosaminoglycans. Once loaded with lipids, macrophages struggle to migrate and egress out of the lesion, contributing to necrotic core expansion. Fourth, due to increased sensitivity to cell death induced by the elevated lipid environment and/or insufficient insulin signaling, and, fifth, reduced efferocytosis capacity, lesions progress faster under diabetic conditions than under non-diabetic conditions and develop expanded necrotic cores. Similar mechanisms are involved in mediating the detrimental effects of diabetes on lesion regression.

Many pieces of this puzzle remain to be found. Although monocytes and macrophages are present in all stages of atherosclerosis and their function is altered by diabetes, a major question is whether the myeloid cell compartment could be successfully targeted for prevention of diabetes-accelerated atherosclerosis and CVD. The CANTOS trial was the first trial to target inflammation and produce a protective effect on CVD. Such an approach might be feasible and successful in people with diabetes and heightened inflammation. Furthermore, based on the research available to date, new therapies that target apolipoproteins, such as APOC3, would be predicted to be effective in preventing CVD in patients with diabetes and elevated levels of APOC3. Other important questions for future research include the mechanisms whereby diabetes induces adverse effects in myeloid cells. For example, what exactly goes awry in myeloid cell lipid handling in the setting of diabetes, and what is the mechanism whereby lipids accumulate in these cells? What is the role of inflammatory changes vis-à-vis lipid over-accumulation? What changes are critical for necrotic core expansion and CVD events? Do different myeloid cell populations respond differently to the diabetic environment? Some of these important answers are likely to be revealed by new methodology, such as single-cell RNA-sequencing, proteomics, metabolomics and lipidomics.

## Author Contributions

JK and C-CH wrote the manuscript. KB wrote parts of the manuscript and edited the full manuscript.

### Conflict of Interest

The authors declare that the research was conducted in the absence of any commercial or financial relationships that could be construed as a potential conflict of interest.
